# PB.31: B3 lesions and vacuum-assisted biopsy: a national survey to gauge current practice

**DOI:** 10.1186/bcr3531

**Published:** 2013-11-08

**Authors:** CL Strachan, A Shaaban, K Horgan, N Sharma

**Affiliations:** 1 St James University Hospital, Leeds, UK

## Introduction

Breast screening detects asymptomatic abnormalities which occasionally on biopsy are classified indeterminate (B3). Such lesions have malignant potential and traditionally are subject to open diagnostic excision biopsy. Vacuum-assisted biopsy (VAB) offers larger representative tissue sampling, and may act as a therapeutic measure completely excising the lesion. The use of VAB in the NHSBSP varies widely. Currently there are no relevant national guidelines to streamline practice.

## Methods

A survey was sent to 80 screening units throughout England, comprising seven questions concerning the use of VAB for B3 lesions.

## Results

Fifty-four responses (67.5%) were received. Twenty-two per cent of units do not perform VAB, 55% perform first-line and 77.8% second-line VAB. For B3 lesions *without *atypia, 68% would proceed to second-line VAB whilst 25% advocate open diagnostic excision following initial (14G) core. Management of B3 lesions *with *atypia was more discordant, with the majority of units opting for second-line VAB for FEA, ALH and LCIS, and second-line diagnostic excision for radial scars, ADH (atypical intraductal proliferation) and papillomas with atypia. Following first-line VAB, most units would proceed to diagnostic excision rather than second-line VAB.

## Conclusion

Management of B3 lesions varies significantly across screening units. There is no concordance in the use of VAB for diagnosis or management of B3 lesions. Whilst there is a trend toward second-line VAB for atypias, significant numbers still opt for diagnostic excision. Consensual national guidelines to standardise and guide management would provide equity of care for this difficult management entity.

